# Age-related changes in grey and white matter structure throughout adulthood

**DOI:** 10.1016/j.neuroimage.2010.03.004

**Published:** 2010-07-01

**Authors:** Antonio Giorgio, Luca Santelli, Valentina Tomassini, Rose Bosnell, Steve Smith, Nicola De Stefano, Heidi Johansen-Berg

**Affiliations:** a Centre for Functional MRI of the Brain, University of Oxford, Oxford, UK; b Neurology and Neurometabolic Unit, Department of Neurological and Behavioural Sciences, University of Siena, Italy; c Department of Neuroscience, University of Padua Medical School, Padua, Italy

## Abstract

Normal ageing is associated with gradual brain atrophy. Determining spatial and temporal patterns of change can help shed light on underlying mechanisms. Neuroimaging provides various measures of brain structure that can be used to assess such age-related change but studies to date have typically considered single imaging measures. Although there is consensus on the notion that brain structure deteriorates with age, evidence on the precise time course and spatial distribution of changes is mixed. We assessed grey matter (GM) and white matter (WM) structure in a group of 66 adults aged between 23 and 81. Multimodal imaging measures included voxel-based morphometry (VBM)-style analysis of GM and WM volume and diffusion tensor imaging (DTI) metrics of WM microstructure. We found widespread reductions in GM volume from middle age onwards but earlier reductions in GM were detected in frontal cortex. Widespread age-related deterioration in WM microstructure was detected from young adulthood onwards. WM decline was detected earlier and more sensitively using DTI-based measures of microstructure than using markers of WM volume derived from conventional T1-weighted imaging.

## Introduction

Studying the distribution and time course of alterations that occur in the normal brain with ageing is important for understanding the mechanisms leading to these changes and for better characterising neurological disorders whose risk increases with advancing age (e.g., dementia). Magnetic resonance imaging (MRI) studies have been particularly important in this regard (Anderson et al., 2005; Galluzzi et al., 2008; Sullivan and Pfefferbaum, 2007).

The advent of quantitative techniques based on the analyses of MRI structural data, such as Voxel-Based Morphometry (VBM) and volumetric analyses, has allowed a sensitive detection of regional patterns of grey matter (GM) and white matter (WM) volume loss. GM volume reduction begins in early adulthood and continues approximately linearly throughout adulthood (Ge et al., 2002; Lehmbeck et al., 2006; Sowell et al., 2003; Walhovd et al., 2005). By contrast, total WM volume change is characterised by a nonlinear relationship with age, with an increase until approximately the fifth decade of life and a decline thereafter (Bartzokis et al., 2001; Pfefferbaum et al., 1994; Salat et al., 1999; Sowell et al., 2003; Walhovd et al., 2005).

More recently, diffusion tensor imaging (DTI) has been used to quantify alterations in WM microstructure during ageing (Abe et al., 2002; Ardekani et al., 2007; Bhagat and Beaulieu, 2004; Head et al., 2004; Madden et al., 2004; O'Sullivan et al., 2001; Ota et al., 2006; Pfefferbaum et al., 2005, 2000; Pfefferbaum and Sullivan, 2003; Salat et al., 2005a,b, 2008; Sullivan et al., 2006; Virta et al., 1999). By fitting a model (the diffusion tensor) to the MRI diffusion data at each brain voxel, this technique allows us to estimate important parameters such as fractional anisotropy (FA: a measure of the degree of diffusion directionality), mean diffusivity (MD), and the three diffusivities (parallel and two perpendicular) of the diffusion tensor (Basser, 1995; Pierpaoli and Basser, 1996). Diffusion metrics are sensitive to age-related changes, but in some cases the correspondence between changes detected using DTI and conventional volume or VBM measurements is not clear.

In the current study we used VBM and DTI measures of GM and WM to study age-related effects on brain structure in a population of healthy adult subjects. We aimed to determine the timing and spatial distribution of age-related changes using a range of volumetric and DTI-based measures.

## Materials and methods

MRI data were acquired in a group of 66 healthy subjects (31 males, 35 females, median age = 36.7 years, range = 23.0-81.6 years, all right-handed). We tested for linear and nonlinear relationships between brain structure and age (see below) and also performed subgroup analyses for which we divided subjects into young adults (YA) (n = 37, 16 males, 21 females, median age = 29.1, range = 23.0-40.2 years), middle-aged adults (MA) (n = 19, 9 males, 10 females, median age = 48.0, range = 41.0-59.6 years) and older adults (OA) (n = 10, 6 males, 4 females, median age = 67.9, range = 60.0-81.6 years). The ages chosen for defining the different age subgroups are broadly consistent with previous studies in YA (Giorgio et al., 2008; McLaughlin et al., 2007; O'Sullivan et al., 2001; Pfefferbaum et al., 2005; Sullivan et al., 2006), MA (Salat et al., 2005a,b) and OA (McLaughlin et al., 2007; Salat et al., 2005b).

None of the participants had a history of psychiatric or neurological disease or substance abuse. On MRI they did not show overt abnormalities such as infarct, vascular malformation or tumour and none of them had WM lesions. Informed written consent was obtained from all participants according to ethical approval from the Oxfordshire Research Ethics Committee C.

## Data acquisition

Scans were obtained on a 1.5T Siemens Sonata MR scanner using a standard single-channel head coil with a maximum gradient strength of 40 mT m^-1^. For the DTI data, two sets of echo-planar images (EPI) of the whole head were acquired (TR = 8500 ms; TE = 80 ms; 53 × 2.5 mm axial slices; in-plane resolution = 2.5 × 2.5 mm^2^). Each set comprised 3 non-diffusion-weighted and 60 diffusion-weighted images acquired with a b-value of 1000 s mm^-2^ uniformly distributed across 60 gradient directions. A T1-weighted image was also acquired in each participant for a VBM-style analysis and image registration (3D FLASH, TR = 12 ms, TE = 5.6 ms, 1 mm isotropic voxels, matrix = 256 × 256 × 208, 3 averages, elliptical sampling, coronal orientation). The absence of WM lesions was confirmed on both non-diffusion- weighted and T1-weighted images.

## Data analysis

### Voxel-based morphometry analysis

We employed an ‘optimised’ VBM-style protocol (Good et al., 2001) using FSL (FMRIB Software Library v4.1, www.fmrib.ox.ac.uk/fsl; (Smith et al., 2004)) tools for brain extraction (Smith, 2002) and tissue segmentation (Zhang et al., 2001). FMRIB's Nonlinear Image Registration Tool (FNIRT) (Andersson et al., 2007a,b) was used to spatially register the native images to a standard-space template. The aim was to assess correlations of regional GM and WM volumes with age. The raw T1-weighted images were first segmented to form images representing partial volume estimates of each tissue class (Zhang et al., 2001). For the analysis of GM, the native GM images were affinely registered to the ICBM-152 GM template, mirror images were created flipping the images across the midline and a left–right symmetric study-specific GM template was created from the average of these images. The native GM images were then nonlinearly transformed to this template using FNIRT. The signal in each voxel of the transformed images was modulated by the Jacobian determinant of the nonlinear component of the warp field. This modulation adjusts the signal in each voxel to reflect (or compensate for) the amount of contraction or enlargement due to the nonlinear component of the spatial transformation. All modulated normalised GM volume images were smoothed with an isotropic Gaussian kernel with a sigma of 3.5 mm (∼ 8 mm FWHM). To test for linear and nonlinear (quadratic) local correlations between changes in GM volume and age, a regression analysis was performed using age and age^2^ as regressors. To compare GM volumes between the different age subgroups, unpaired t-tests were used. Statistical inference on all analyses was performed using the *randomise* programme within FSL, which performs permutation testing (5000 permutations) (Nichols and Holmes, 2002). Thresholding was carried out using TFCE (Threshold-Free Cluster Enhancement), a method for finding significant clusters in MRI data without having to define them in a binary way (Smith and Nichols, 2009). Clusters were assessed for significance at p < 0.05, fully corrected for multiple comparisons across space. Anatomical locations of the significant GM clusters were determined by reference to the Harvard-Oxford cortical structural atlas integrated into FSLView (part of FSL).

For the WM we used a protocol similar to that of the GM (see above). All modulated normalised WM volume images were smoothed with an isotropic Gaussian kernel with a sigma of 4 mm (∼ 10 mm FWHM). Anatomical location of the significant WM clusters were determined by reference to an atlas of human WM anatomy (Wakana et al., 2004) integrated into FSLView.

The different levels of smoothing for GM and WM were chosen to be compatible with previous studies (e.g., (Giorgio et al., 2010; Paus et al., 1999; Sowell et al., 1999)).

## Diffusion data analysis

Diffusion MRI data were pre-processed using DTIFit within the FMRIB Diffusion Toolbox (FDT, part of FSL). Images were corrected for eddy currents and head motion by using affine registration to the non-diffusion volumes (b = 0). Data were averaged across the two acquisitions to improve signal-to-noise ratio. Images of FA, MD, diffusivity parallel (λ_1_) and perpendicular ((λ_2_ + λ_3_)/2) to the principal diffusion direction were obtained. Voxelwise correlations of FA with age and FA comparisons between the different age subgroups were assessed using TBSS (part of FSL (Smith et al., 2006)). FA images were first nonlinearly (using FNIRT) registered to a high resolution standard-space average of 58 well aligned good quality FA images from healthy subjects and then averaged. The average of the data was thinned to create a ‘skeleton’ of WM, representing the tracts common to all subjects. This WM tract skeleton was thresholded at FA > 0.2, resulting in a skeleton with 114884 1 × 1 × 1 mm^3^ WM voxels, corresponding to approximately two thirds of the WM voxels of the whole skeleton. TBSS then projects each subject's FA data onto the mean WM tract skeleton. The highest FA value on a line perpendicular to each point on the skeleton in each subject (which should correspond to the local tract centre value) is then projected onto the mean WM tract skeleton. Next, values of MD were mapped onto the skeleton by using the projection vectors from each subject's FA-to-skeleton transformation. The FA and MD data were analysed using the same models as for the GM and WM volume analyses described above in order to examine the linear and nonlinear (quadratic) correlation of FA (and MD) with age and the FA (and MD) comparisons between the different age subgroups. Statistical inference was carried out using TFCE thresholding in randomise (5000 permutations). Clusters were thresholded at p < 0.05, fully corrected for multiple comparisons across space. The anatomical location of the significant clusters was determined by reference to a fibre tract-based atlas of human WM anatomy (Wakana et al., 2004) integrated into FSLView. In addition to the FA and MD data used for analysis on the WM skeleton in each participant (see above), the parallel and perpendicular diffusivity data from the same voxels were also processed using the TBSS protocol.

For clusters showing significant correlation of FA (and MD) with age using TBSS, we calculated mean FA, MD, and parallel and perpendicular diffusivity values across all voxels within the significant clusters and plotted these against age to help visualize the distribution of values.

## Results

### Correlations of grey and white matter volume with age across the whole group

**Fig. 1:**
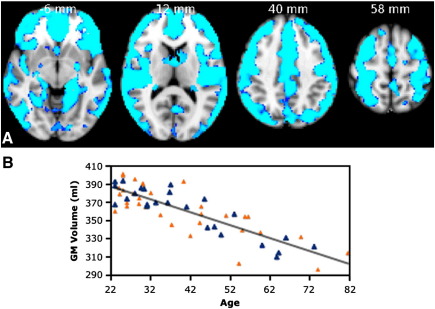
VBM-style analysis of GM changes with age. (A) Coloured voxels show regions demonstrating significant negative correlations between GM volume and age (p < 0.05, fully corrected for multiple comparisons across space). Clusters are overlaid on the MNI152 template brain. Images are shown in radiological convention. (B) Plot to illustrate relationship between age and mean GM volume across all significant voxels. The orange triangles represent female subjects.

VBM-style analyses were run to assess associations of GM and WM volumes with age. Most cortical regions, with the exception of occipital pole, showed an extensive linear negative correlation of GM volume with age (Fig. 1A), especially in the left superior frontal gyrus (SFG), right middle frontal gyrus (MFG), left postcentral gyrus, right superior parietal lobule and right lateral occipital cortex (Table 1). Negative correlation between volume and age was also found in deep GM structures such as the caudate nucleus, pallidum, amygdala and hippocampus bilaterally. The correlation between volume averaged across all the GM clusters and age was r = -0.84, p < 0.001 (Fig. 1B). There were no significant linear positive or nonlinear relationships between GM volume and age.

**Fig. 2:**
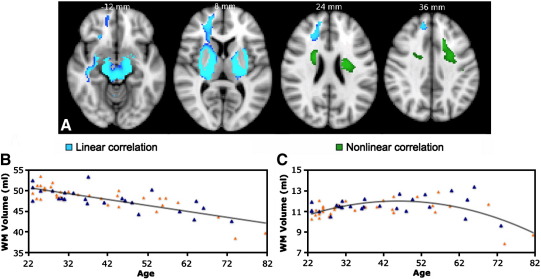
VBM-style analysis of WM changes with age. (A) Coloured voxels show regions where WM volume shows a significant linear (blue) or non-linear (green) relationship with age (p < 0.05, fully corrected for multiple comparisons across space). Clusters are overlaid on the MNI152 template brain. Images are shown in radiological convention. (B, C) Plots to illustrate relationship between age and mean WM volume across all voxels showing a significant linear (B) or nonlinear (C) relationship with age. The orange triangles represent female subjects.

The most significant linear negative correlation of WM volume with age were found on the right hemisphere in the anterior thalamic radiations, anterior limb of the internal capsule (ALIC), cerebral peduncle (CP), cerebellum and bilaterally in the external capsule (EC) (Fig. 2A; Table 2). The correlation between volume averaged across all these WM clusters and age was r = -0.79, p < 0.001 (Fig. 2B). No significant linear positive correlation between WM volumes and age were found. Significant nonlinear (quadratic) relationships between WM volume and age were found in the left superior longitudinal fascicle (SLF) and superior corona radiata (SCR) bilaterally (Figs. 2A and C; Table 3).

Correlations of GM and WM volumes with age were not different between males and females.

### Correlations of DTI metrics with age across the whole group

**Fig. 3:**
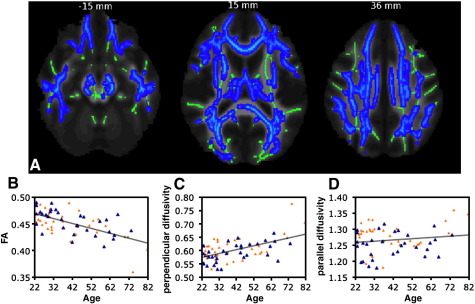
TBSS analysis of changes in FA with age. (A) Blue voxels show WM regions where FA shows a significant negative linear relationship with age (p < 0.05, fully corrected for multiple comparisons across space). Voxels are overlaid on the WM skeleton (in green) and the group mean FA image (greyscale). Images are shown in radiological convention. (B, C, D) Plots to illustrate the relationship between age and mean FA (B), perpendicular diffusivity (C) and parallel diffusivity (D) across all voxels showing a significant linear relationship between age and FA. The orange triangles represent female subjects.

TBSS analyses of diffusion parameters were carried out to test for age-related effects on FA, MD and diffusivities. Linear negative correlations of FA with age were found in most WM regions (Fig. 3A) and were highest in the SCR bilaterally (Table 4). Region-of-interest (ROI) analysis of these clusters showed that correlation between mean FA and age was r = -0.61, p < 0.001 (Fig. 3B). There were no significant linear positive and nonlinear (quadratic) relationships between FA and age.

To test whether the linear negative correlation of FA with age was due to significant associations of parallel and/or perpendicular diffusivity, we computed the mean of these diffusivities across voxels within all significant clusters and tested for correlations with age. This analysis revealed significant correlations of perpendicular (r = 0.54, p < 0.001; Fig. 3C) but not parallel (r = 0.14, N.S.; Fig. 3D) diffusivity with age.

**Fig. 4:**
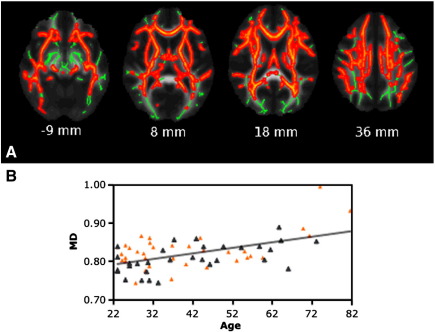
TBSS analysis of changes in MD with age. (A) Red voxels show WM regions where MD shows a significant positive linear relationship with age (p < 0.05, fully corrected for multiple comparisons across space). Voxels are overlaid on the WM skeleton (in green) and the group mean FA image (greyscale). Images are shown in radiological convention. (B) Plot to illustrate the relationship between age and mean MD across all voxels showing a significant linear relationship between age and MD. The orange triangles represent female subjects.

MD showed a linear positive correlation with age in many WM regions, with the posterior regions being less extensively affected (Fig. 4A). This correlation was highest in the SCR bilaterally, in the right SLF and inferior fronto-occipital fascicle (IFOF) (Table 5). The correlation between MD values averaged across all these WM clusters and age was r = 0.52, p < 0.001 (Fig. 4B). No significant linear negative or nonlinear relationships of MD with age were found.

Correlations of FA and MD with age were not different between males and females.

### Comparisons of grey and white matter volumes and diffusion metrics between age subgroups

In order to identify the phases of adulthood when changes were occurring, we performed comparisons of structural measures between YA, MA and OA. However, these analysis were relatively limited by the presence of a lower number of subjects in the MA and especially OA subgroups with respect to YA subgroup.

**Fig. 5:**
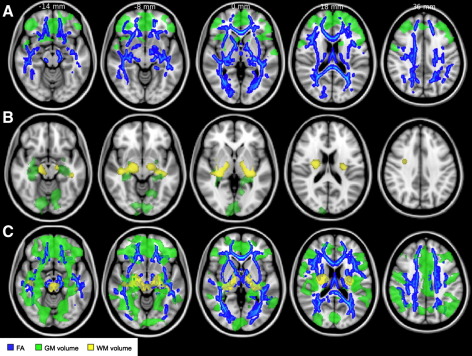
Subgroup analysis of GM volume and WM volume and FA. (A) Comparisons between the YA and MA subgroups revealed reductions in frontal GM volume (green) and in FA throughout the WM skeleton (blue). (B) Comparisons between the MA and OA subgroups revealed later reductions in posterior GM volume (green) and in WM volume at the level of internal capsule and corona radiata (yellow). (C) Comparisons between the YA and OA subgroups revealed widespread reductions in GM volume (green) and FA (blue) and more localised reductions in WM volume (yellow). All analyses were thresholded at p < 0.05, fully corrected for multiple comparisons across space. See text for abbreviations. Images are shown in radiological convention.

Subgroup comparisons based on the VBM-style analysis of GM volume broadly revealed early frontal reduction followed by a later more widespread reduction. Specifically, reduction in GM volume was found in the MA compared to YA bilaterally in the frontal lobe, including the caudate nucleus and, to a lesser extent, in the temporal lobe (Fig. 5A, green; Table 6, supplemental material). A lower GM volume in the OA compared to MA was present bilaterally in the lingual gyrus, and on the right in the occipital fusiform gyrus and hippocampus (Fig. 5B, green; Table 7, supplemental material). OA showed a widespread reduction in GM volume compared to YA in several regions (Fig. 5C, green), especially in the MFG bilaterally, left SFG, right supplementary motor cortex, right precentral gyrus and left cerebellum (Table 8, supplemental material).

Subgroup comparisons of the VBM-style analysis of WM revealed that reduction in WM volume became apparent only in late adulthood. Specifically, we found no significant differences in WM volume between YA and MA whereas OA showed a reduction in WM volume compared to MA on the right hemisphere in the SLF, EC, posterior limb of the internal capsule (PLIC), fornix/stria terminalis, IFOF, bilaterally in the CP, and on the left hemisphere in the retro-lenticular part of the internal capsule (IC) and inferior longitudinal fascicle (ILF) (Fig. 5B, yellow; Table 9, supplemental material). Similar WM regions showed a significant volume reduction in OA compared to YA (Fig. 5C, yellow), with the highest differences located in the left sagittal stratum, CP bilaterally and left cerebellum (Table 10, supplemental material).

**Fig. 6:**
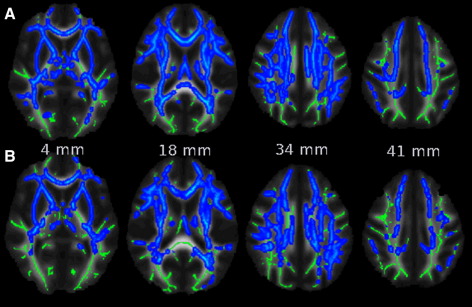
Subgroup analysis of WM MD. (A) Comparisons between the YA and OA subgroups revealed MD increase in OA (blue) in most of the WM regions. (B) Comparisons between the MA and OA subgroups revealed MD increase in OA (blue) in most of the WM regions. All analyses were thresholded at p < 0.05, fully corrected for multiple comparisons across space. See text for abbreviations. Images are shown in radiological convention.

Subgroup comparisons of diffusion measures analysed using TBSS revealed that widespread FA reductions occur from middle age. Specifically, MA had lower FA than YA in many WM regions (Fig. 5A, blue), and this difference was highest in the right SCR, right body of the corpus callosum and left SLF (Table 11, supplemental material). OA had also lower FA than YA in many WM regions (Fig. 5C, blue), especially in the SCR bilaterally, right SLF and left forceps minor (Table 12, supplemental material). No significant FA differences were found between MA and OA (Fig. 5B). Interestingly, subgroup analysis suggested that MD increase becomes apparent later than FA reductions. Specifically, MD was higher in OA compared to YA in many WM regions (Fig. 6A), and this difference was highest in the right SCR, left SLF and right IFOF (Table 13, supplemental material). Compared to MA, MD in OA was higher in many WM regions (Fig. 6B), especially in the right SLF and IFOF (Table 14, supplemental material). No significant MD differences were found between YA and MA.

## Discussion

We scanned 66 healthy subjects aged between 23 and 81 years and found evidence for a widespread linear negative association of GM volumes and a more localised negative association of WM volumes with age. Furthermore, we found that FA, a putative marker of integrity of WM microstructure, was negatively associated with age in many fibre tracts, mainly due to a positive association of diffusivity perpendicular to the main axis of the fibre tracts. MD, which reflects overall diffusivity, showed a positive association with age in many WM regions, and this was less extensive in the occipital lobe. Each of these patterns of change, and the putative mechanisms underlying them, are discussed in detail below.

### Grey matter volume

In our study most cortical and deep GM regions showed a linear negative association between volume and age. These findings are broadly in line with previous studies of similar cohorts (Good et al., 2001; Jernigan et al., 2001; Pfefferbaum et al., 1994; Raz et al., 1997; Resnick et al., 2000; Salat et al., 2004; Sowell et al., 2003; Sullivan et al., 1995, 2004; Walhovd et al., 2005), although the volume reductions we detected are even more widespread than some previous reports that have found preservation of volume in specific structures such as amygdala and hippocampus (Good et al., 2001; Sullivan et al., 1995).

There are a number of potential cellular processes that could explain these volume reductions in GM. In early adulthood, elimination of neurons and synapses (Ge et al., 2002; Webb et al., 2001) can contribute, whereas in middle and late adulthood volume reductions could be due to shrinkage of large neurons (Peters et al., 1998; Terry et al., 1987), or rarefaction in the GM microvasculature, resulting in a loss of neurons (Farkas and Luiten, 2001; Riddle et al., 2003). In addition, GM volume reductions inferred from T1-weighted images may be apparent, arising not only from genuine decreases in neuronal volume but also from changes in myelination, affecting the intensity of the MR signal (Sowell et al., 2003). This may be a plausible mechanism in early adulthood because of ongoing processes of myelination (Yakovlev and Lecours, 1967) but is less likely to play a role in later adulthood. This last hypothesis may explain why different ageing trajectories have been previously found in different cortical areas, appearing linear in visual, auditory and limbic cortices, which complete myelination early in life, and nonlinear or more complex in associative frontal and parietal cortices and posterior temporal cortex, which continue myelination into adulthood (Sowell et al., 2003).

### White matter volume

The total WM volume follows a nonlinear inverted “U-shaped” relationship with age, slightly increasing during the early adulthood, consistent with the notion of an ongoing maturation of the WM beyond adolescence, and then reaching the peak in the fourth decade (Ge et al., 2002; Sowell et al., 2003; Walhovd et al., 2005).

Using a VBM-style analysis, we found linear negative association of WM volume with age especially in the external capsule bilaterally, and in the right anterior limb of the internal capsule, anterior thalamic radiations, cerebral peduncle and cerebellum. Significant nonlinear (quadratic) relationships between WM volume and age were present in the superior corona radiata bilaterally and in the left superior longitudinal fascicle. These findings seem to contrast with those of a recent study on 84 normal subjects aged 13-70 years (Pagani et al., 2008), where linear negative associations between volume an age were found in the fronto-parietal lobes (corona radiata, anterior cingulum, fornix) and left cerebellar peduncle and nonlinear relationships with age in the genu of the corpus callosum and in the right deep temporal association fibres. The presence of different areas where WM volume is related to age, found by the two studies, may be due to the fact that Pagani et al. (2008) included younger subjects and used a DTI-based technique, and not VBM, to assess white matter fibre volumes.

Our findings suggest that both heavily myelinated fibres (e.g., corticospinal tracts, located in the posterior limb of the internal capsule) and thinly myelinated fibres (e.g., association fibres of the frontal lobe) are affected in the same way by the pathogenic mechanisms underlying the ageing process. The involvement of the motor system during ageing is confirmed in the current study by the fact that volume in precentral gyrus, where that system is expected to originate, showed a significant inverse correlation with age and by the findings of previous histological studies, where an age-related decrease in the number of pyramidal fibres in the spinal cord and a thinning of precentral gyrus were reported (Salat et al., 2004; Terao et al., 1994).

WM atrophy could be a result of the ageing process *per se* or a consequence of the effects of WM lesions, which may be present in the neurologically asymptomatic elderly population (Enzinger et al., 2005; Pagani et al., 2008; Rovaris et al., 2003). However, none of our subjects had the presence of WM lesions and, further, a previous study found no overlap between regions showing age-related atrophy of WM fibres and the presence of WM lesions (Pagani et al., 2008).

Overall, we demonstrated here that the loss of WM integrity over adulthood and ageing might be explained in the corticospinal tract by WM atrophy.

### Diffusion metrics in white matter

In addition to the VBM-style analysis of WM volume described above, we also tested for age-related change in DTI metrics of WM microstructure. We found widespread linear negative associations of FA with age, consistent with previous reports (Ardekani et al., 2007; Head et al., 2004; Madden et al., 2004; O'Sullivan et al., 2001; Pfefferbaum and Sullivan, 2003; Salat et al., 2005a, 2010).

When comparing the different age subgroups, both middle-aged (41-59 years) and older (60-81 years) adults showed a significant widespread FA decrease compared to young adults (23-40 years) whereas middle-aged and older adults were not significantly different from each other, suggesting that the loss of directionality in WM fibres becomes apparent during the transition from early to middle adulthood. Again, this is in line with the study by Salat et al. (Salat et al., 2005a), where FA differences between young adults (21-37 years) and older adults (65-76 years) were apparent by middle adulthood (42-59 years). In the current study, the presence of widespread FA reductions in the transition from young to middle adulthood was demonstrated earlier than the volume reductions shown by our WM VBM-style analysis and earlier than the existing volumetric literature on WM (Bartzokis et al., 2001; Ge et al., 2002; Sowell et al., 2003; Walhovd et al., 2005). This regional decline in FA with early ageing occurs in the absence of significant regional increase in FA elsewhere, suggesting that middle adulthood may be the time when the transition from development to ageing in white matter fibre coherence takes place.

The findings of our study challenge the idea that early myelinating posterior regions are less susceptible to age-related change than late myelinating regions of frontal lobe white matter. Although previous DTI studies have provided some support for this idea, i.e. greater age-related FA decline in frontal compared to posterior WM regions (Abe et al., 2002; Ota et al., 2006; Pfefferbaum et al., 2005; Salat et al., 2005a,b; Sullivan et al., 2006), our results are more closely in line with studies where a decrease in FA was found not only in regions of the frontal lobe but also in more posterior regions such as the splenium of the corpus callosum and posterior limb of the internal capsule (Ardekani et al., 2007; Furutani et al., 2005; Salat et al., 2005a).

Our results also demonstrate that the two major tracts connecting limbic structures in Papez circuit (i.e., fornix and cingulum) are both affected by ageing. The involvement of the fornix in our cohort of subjects is also confirmed by the fact that volume of hippocampus, a GM structure connected by the fornix, showed a significant inverse correlation with age. However, these results appear to conflict with a recent study in subjects aged 18 to 88 years, where cingulum but not fornix appeared resistant to ageing (Stadlbauer et al., 2008).

In our study, MD showed a spatial pattern of associations with age broadly similar to FA, although it seemed that posterior regions of the WM were less extensively affected. The comparison across different age subgroups showed that, in contrast to FA changes, changes in WM MD appear as a late ageing phenomenon, consistent with a previous study which used slightly different age ranges for the two groups (Ardekani et al., 2007).

The assessment of parallel and perpendicular diffusivity may enhance the specificity of DTI findings and shed light on the mechanisms underlying FA changes. Changes in parallel and perpendicular diffusivity have been related to axon and myelin damage, respectively, in mouse models of multiple sclerosis (Budde et al., 2007a,b; Song et al., 2005; Sun et al., 2007; Wu et al., 2007), dysmyelination (Song et al., 2002) and retinal ischemia in the optic nerve (Song et al., 2003). Moreover, perpendicular diffusivity, together with FA and MD, was shown to be a robust predictor of myelin content in *post-mortem* human brain, prior to and after fixation (Schmierer et al., 2008). However, the degree to which these relationships hold in the healthy living human brain remains to be determined. Nevertheless, interrogating how the different diffusivities contribute to observed FA changes provides complementary information. In the current study, we found that negative correlation of FA with age was mainly driven by positive correlation of perpendicular diffusivity, whereas parallel diffusivity only showed a slight non-significant positive correlation with age. These findings are in line with other recent studies testing the tensor diffusivities during ageing (Bastin et al., 2009; Bhagat and Beaulieu, 2004; Ota et al., 2006; Salat et al., 2010; Stadlbauer et al., 2008; Sullivan et al., 2010; Vernooij et al., 2008) and may be explained by the presence of degenerative changes in the myelin sheaths or, alternatively, of a reduced fibre organisation or “packing” (i.e., a decrease in the number of axons) within WM tracts.

In our study, FA changes overlap with regions showing evidence for WM atrophy (i.e., reductions in WM volume using VBM-style analysis) in the forceps minor, internal and external capsules, cerebral peduncle and temporal association fibers, consistent with previous reports showing similar changes in FA and VBM measures of WM (Vernooij et al., 2008). At the level of the superior corona radiata, however, out data suggest that this relationship seems to be present only after 50 years of age. Further, in all others areas loss of WM microstructure was detected in the absence of significant WM changes on VBM-style analysis. This suggests that DTI can provide more sensitive information on ageing-related WM degeneration compared to volume metrics.

Our study benefited from some methodological advances compared to previous DTI studies investigating ageing-related FA changes. First, data were acquired using 60 diffusion directions, thus providing increased power and higher angular resolution compared to some previous investigations. Second, we used a technique for sensitive and robust voxelwise analysis of DTI data (Smith et al., 2006) as well as an improved approach to statistical thresholding which enhances all cluster-like structures present in an image avoiding the need for the arbitrary initial cluster-forming threshold (Smith and Nichols, 2009).

In studies on the ageing brain, the quality of image registration is an important issue, especially as it applies to small structures such as the hippocampus. Indeed, an age-related volume decline of such structures may represent an artifact of distorted shape from adjacent, expanding fluid-filled spaces rather than a real atrophy process. However, as registration was accurately checked, we do not believe that results of this study are due to misregistration.

However, there are some limitations to this study. First, we had fewer subjects in the older age groups, and this might have underpowered the subgroup comparison analyses. Second, the study has a cross-sectional design and therefore, because it contains different cohort of subjects in different age groups, it may be less sensitive to brain changes across the lifespan than a longitudinal study. Third, we recognise that age clustering in this study is arbitrary. However, as we mention in the Methods section, grouping selection was broadly in line with previous studies. In addition, we deliberately included complementary analyses (i.e., testing for correlations across the whole age range) that were not dependent on this grouping. Finally, after performing residual analysis of all plotted measures in the different regression analyses, we found no clear evidence for heteroscedasticity and no outliers in the VBM or FA analyses and only one outlier in the MD analysis (data not shown). However, it should be noted that regression analysis is relatively robust to homoscedasticity and small-to-moderate violations of homoscedasticity have only minor impact on regression estimates (Fox, 2005).

In conclusion, we assessed a number of measures of brain structure to characterise the spatial and temporal pattern of changes that occur in both GM and WM with normal ageing. Reductions in GM volume appear in middle age and then become more widespread into older adulthood. Widespread age-related decline in WM microstructure occurs earlier and can be detected more sensitively using DTI-based measures of microstructure than using markers of WM volume derived from conventional T1-weighted imaging. The observed patterns and dynamics of normal age-related changes may have important implications for future studies on chronic neurological conditions that show an impact of age on disease onset, course and progression, such as Alzheimer's disease, Parkinson's disease and multiple sclerosis (Baquer et al., 2009; Reeve et al., 2008; Trojano et al., 2002). The application of these novel MRI techniques may be useful for furthering our understanding of pathologic substrates underlying such neurological conditions, for charting their evolution and monitoring response to treatment. Finally, the study of age-related brain changes may potentially benefit from the use of high field scanner MRI acquisition, which provides increased signal-to-noise ratio and so opens possibilities for greater spatial resolution, although issues related to field inhomogeneities and increased chemical shift (Pagani et al., 2007) deserve further investigation.

## Figures and Tables

**Table 1:** Local maxima within each significant cluster showing significant (corrected p < 0.05) negative correlations between grey matter volume and age. The grey matter clusters are ordered by decreasing Y coordinates of their local maxima.

Cluster no. (voxel no.)	Side	MNI			t-statistic
Region	X	Y	Z
1 (84498)					
Superior frontal gyrus	L	-24	14	52	2.78
Middle frontal gyrus	R	32	10	52	2.84
Superior frontal gyrus	L	-24	6	56	2.89
Postcentral gyrus	L	-34	-28	54	2.91
Superior parietal lobule	R	20	-54	62	2.79
Lateral occipital cortex	R	14	-60	62	2.85

**Table 2:** Local maxima within each significant cluster showing significant (corrected p < 0.05) linear negative correlations between white matter volume and age. The white matter clusters are ordered by decreasing Z coordinates of their local maxima.

Cluster no. (voxel no.)	Side	MNI			t-statistic
Region	X	Y	Z
*1 (8110)*					
Anterior thalamic radiation	R	12	-10	16	4.58
Anterior limb of internal capsule	R	20	16	0	3.85
External capsule	R	36	-8	0	4.98
R	34	6	-2	4.88
L	-32	-2	-2	4.78
L	-34	-16	-2	4.48
Cerebral peduncle	R	18	-22	-14	4.34

*2 (89)*					
Cerebellum	R	26	-46	-48	3.08
R	22	-44	-50	3.57

**Table 3:** Local maxima within each significant cluster showing significant (corrected p < 0.05) nonlinear relationships between white matter volume and age. The white matter clusters are ordered by decreasing Z coordinates of their local maxima.

Cluster no. (voxel no.)	Side	MNI			t-statistic
Region	X	Y	Z
*1 (1112)*					
Superior longitudinal fascicle	L	-34	-8	42	3.37
L	-14	16	40	4.94
L	-44	-18	30	3.82
L	-22	-8	24	3.76

*2 (344)*					
Superior corona radiata	R	22	2	26	4.28

**Table 4:** Local maxima within each significant cluster showing significant (corrected p < 0.05) linear negative correlations between white matter FA and age. The white matter clusters are ordered by decreasing Z coordinates of their local maxima.

Cluster no. (voxel no.)	Side	MNI			t-statistic
Region	X	Y	Z
*1 (60191)*					
Superior corona radiata	R	13	-17	61	2.20
R	13	-19	61	2.13
R	14	-12	58	1.97
Anterior corona radiata	R	12	4	57	1.77
Superior corona radiata	R	15	-10	57	2.01
L	-17	-19	56	2.01

**Table 5:** Local maxima within each significant cluster showing significant (corrected p < 0.05) linear positive correlations between white matter MD and age. The white matter clusters are ordered by decreasing Z coordinates of their local maxima.

Cluster no. (voxel no.)	Side	MNI			t-statistic
Region	X	Y	Z
*1 (52079)*					
Superior corona radiata	L	-19	-19	38	2.84
R	20	-8	37	3.14
R	24	-24	37	3.05
Superior longitudinal fascicle	R	48	-33	37	3.12
Superior corona radiata	R	22	0	36	3.22
Inferior fronto-occipital fascicle	R	35	-4	-13	3.04

## Appendix A Supplementary data

Table 6 Local maxima within each significant cluster showing significant (corrected p < 0.05) lower grey matter volume in the middle adult subgroup compared to the young adult subgroup. The grey matter clusters are ordered by decreasing Y coordinates of their local maxima.

Table 7 Local maxima within each significant cluster showing significant (corrected p < 0.05) lower grey matter volume in the old adult subgroup compared to the middle adult subgroup. The grey matter clusters are ordered by decreasing Y coordinates of their local maxima.

Table 8 Local maxima within each significant cluster showing significant (corrected p < 0.05) lower grey matter volume in the old adult subgroup compared to the young adult subgroup. The grey matter clusters are ordered by decreasing Y coordinates of their local maxima.

Table 9 Local maxima within each significant cluster showing significant (corrected p < 0.05) lower white matter volume in the old adult subgroup compared to the middle adult subgroup. The white matter clusters are ordered by decreasing Z coordinates of their local maxima.

Table 10 Local maxima within each significant cluster showing significant (corrected p < 0.05) lower white matter volume in the old adult subgroup compared to the young adult subgroup. The white matter clusters are ordered by decreasing Z coordinates of their local maxima.

Table 11 Local maxima within each significant cluster showing significant (corrected p < 0.05) lower white matter FA in the middle adult subgroup compared to the young adult subgroup. The white matter clusters are ordered by decreasing Z coordinates of their local maxima.

Table 12 Local maxima within each significant cluster showing significant (corrected p < 0.05) lower white matter FA in the old adult subgroup compared to the young adult subgroup. The white matter clusters are ordered by decreasing Z coordinates of their local maxima.

Table 13 Local maxima within each significant cluster showing significant (corrected p < 0.05) higher white matter MD in the old adult subgroup compared to the young adult subgroup. The white matter clusters are ordered by decreasing Z coordinates of their local maxima.

Table 14 Local maxima within each significant cluster showing significant (corrected p < 0.05) higher white matter MD in the old adult subgroup compared to the middle adult subgroup. The white matter clusters are ordered by decreasing Z coordinates of their local maxima
