# Supplementary material for: Age-related changes in grey and white matter structure throughout adulthood
Source: Neuroimage. 2010 Jul 1;51(3-2):943–51. doi: 10.1016/j.neuroimage.2010.03.004 (PMC2896477; doi:10.1016/j.neuroimage.2010.03.004)
Supplement: Table 6 — Local maxima within each significant cluster showing significant (corrected p < 0.05) lower grey matter volume in the middle adult subgroup compared to the young adult subgroup. The grey matter clusters are ordered by decreasing Y coordinates of their local maxima. [file mmc1.doc]

**Supplemental material**

**Table 6. Local maxima within each significant cluster showing significant (corrected p<0.05) lower grey matter volume in the middle adult subgroup compared to the young adult subgroup. The grey matter clusters are ordered by decreasing Y coordinates of their local maxima.**

| *Cluster no. (voxel no.)*  *Region* | *Side* | *MNI*  *X* | *Y* | *Z* | *t-statistic* |
| --- | --- | --- | --- | --- | --- |
| 1 (23267)  Frontal pole      Frontal medial cortex  Paracingulate gyrus  Frontal medial cortex | R  R  L  L  R  R  R | 2  12  -10  -4  10  2  2 | 58  56  56  44  40  40  36 | 18  8  -2  -20  18  -18  -16 | 3.88  3.89  3.99  3.95  3.97  4.02  4.02 |
| 2 (825)  Temporal pole    Temporal fusiform cortex | R  R R R | 38  30  22  26 | 12  4  0  -10 | -48  -40  -50  -44 | 3.32  3.48  4.07  3.61 |
| 3 (262)  Central opercular cortex  Planum temporale | L  L | -64  -58 | -10  -10 | 8  4 | 4.03  3.78 |
| 4 (255)  Inferior temporal gyrus | L  L  L | -52  -62  -52 | -6  -12  -12 | -34  -34  -32 | 3.23  3.64  3.31 |
| 5 (248)  Precentral gyrus  Postcentral gyrus | L  L | -36  -44 | -16  -22 | 54  48 | 3.05  3.16 |

**Table 7. Local maxima within each significant cluster showing significant (corrected p<0.05) lower grey matter volume in the old adult subgroup compared to the middle adult subgroup. The grey matter clusters are ordered by decreasing Y coordinates of their local maxima.**

| *Cluster no. (voxel no.)*  *Region* | *Side* | *MNI*  *X* | *Y* | *Z* | *t-statistic* |
| --- | --- | --- | --- | --- | --- |
| 1 (7331)  Lingual gyrus    Occipital fusiform gyrus        Lingual gyrus | L  R  R  R R R L | -10  12  18  10  14  10  -10 | -66  -84  -86  -88  -88  -88  -90 | -14  -8  -14  -20  -18  -20  -6 | 4.22  4.40  4.50  4.66  4.46  4.66  4.37 |
| 2 (1331)  Parahippocampal gyrus | R R | 24  30 | -20  -32 | -22  -12 | 4.80  4.22 |

**Table 8. Local maxima within each significant cluster showing significant (corrected p<0.05) lower grey matter volume in the old adult subgroup compared to the young adult subgroup. The grey matter clusters are ordered by decreasing Y coordinates of their local maxima.**

| *Cluster no. (voxel no.)*  *Region* | *Side* | *MNI*  *X* | *Y* | *Z* | *t-statistic* |
| --- | --- | --- | --- | --- | --- |
| 1 (72162)  Middle frontal gyrus    Superior frontal gyrus  Middle frontal gyrus  Supplementary motor cortex  Precentral gyrus  Cerebellum | L  L  L  R  R  R  L | -40  -48  -18  30  12  34  -16 | 18  6  4  -2  -4  -24  -72 | 46  48  50  48  56  54  -36 | 2.95  2.99  4.48  3.48  2.97  2.95  4.43 |

**Table 9. Local maxima within each significant cluster showing significant (corrected p<0.05) lower white matter volume in the old adult subgroup compared to the middle adult subgroup. The white matter clusters are ordered by decreasing Z coordinates of their local maxima.**

| *Cluster no. (voxel no.)*  *Region* | *Side* | *MNI*  *X* | *Y* | *Z* | *t-statistic* |
| --- | --- | --- | --- | --- | --- |
| 1 (2036)  Superior longitudinal fascicle  External capsule  Posterior limb of internal capsule  Fornix/Stria terminalis  Inferior fronto-occipital fascicle  Cerebral peduncle | R  R  R  R  R R | 32  32  22  26  40  18 | -4  -8  -12  -28  -24  -16 | 36  10  2  -2  -8  -12 | 3.68  4.50  6.39  5.07  4.39  5.09 |
| 2 (1555)  Internal capsule (retrolenticular)  Cerebral peduncle  Inferior longitudinal fascicle | L  L  L  L | -24  -32  -14  -42 | -22  -22  -16  -30 | 2  -2  -10  -16 | 6.50  6.04  4.56  3.77 |

**Table 10. Local maxima within each significant cluster showing significant (corrected p<0.05) lower white matter volume in the old adult subgroup compared to the young adult subgroup. The white matter clusters are ordered by decreasing Z coordinates of their local maxima.**

| *Cluster no. (voxel no.)*  *Region* | *Side* | *MNI*  *X* | *Y* | *Z* | *t-statistic* |
| --- | --- | --- | --- | --- | --- |
| 1 (4975)  Sagittal stratum  Cerebral peduncle | L  L  R | -36  -18  20 | -28  -18  -16 | -6  -8  -12 | 3.92  4.88  4.73 |
| 2 (19)  Cerebellum | L | -20 | -72 | -22 | 4.11 |

**Table 11. Local maxima within each significant cluster showing significant (corrected p<0.05) lower white matter FA in the middle adult subgroup compared to the young adult subgroup. The white matter clusters are ordered by decreasing Z coordinates of their local maxima.**

| *Cluster no. (voxel no.)*  *Region* | *Side* | *MNI*  *X* | *Y* | *Z* | *t-statistic* |
| --- | --- | --- | --- | --- | --- |
| 1 (42988)  Superior corona radiata    Body of the corpus callosum | R  R  R R  R  R | 18  18  17  17  17  17 | -9  -3  -1  3  5  11 | 40  38  37  35  34  31 | 2.85  2.77  2.81  2.83  2.97  3.01 |
| 2 (744)  Superior longitudinal fascicle | L  L  L  L  L | -37  -42  -39  -44  -36 | -2  -6  -3  -5  -5 | 28  25  24  24  23 | 3.67  3.09  2.75  2.83  2.67 |

**Table 12. Local maxima within each significant cluster showing significant (corrected p<0.05) lower white matter FA in the old adult subgroup compared to the young adult subgroup. The white matter clusters are ordered by decreasing Z coordinates of their local maxima.**

| *Cluster no. (voxel no.)*  *Region* | *Side* | *MNI*  *X* | *Y* | *Z* | *t-statistic* |
| --- | --- | --- | --- | --- | --- |
| 1 (51118)  Superior corona radiata  Superior longitudinal fascicle  Superior corona radiata  Forceps minor | L  R  R R R  R  L | -13  15  14  15  15  20  -15 | -3  11  15  17  19  -13  37 | 56  52  51  50  49  47  -11 | 2.14  2.10  2.91  2.20  2.49  2.29  2.81 |

**Table 13. Local maxima within each significant cluster showing significant (corrected p<0.05) higher white matter MD in the old adult subgroup compared to the young adult subgroup. The white matter clusters are ordered by decreasing Z coordinates of their local maxima**

| *Cluster no. (voxel no.)*  *Region* | *Side* | *MNI*  *X* | *Y* | *Z* | *t-statistic* |
| --- | --- | --- | --- | --- | --- |
| 1 (49109)  Superior corona radiata  Superior longitudinal fascicle  Inferior fronto-occipital fascicle | R  R  R  R  L  L  R | 20  18  18  19  -15  -15  23 | 13  8  10  18  31  33  15 | 40  40  38  37  36  35  -13 | 3.27  3.48  3.05  3.23  3.27  3.08  3.49 |

**Table 14. Local maxima within each significant cluster showing significant (corrected p<0.05) higher white matter MD in the old adult subgroup compared to the middle adult subgroup. The white matter clusters are ordered by decreasing Z coordinates of their local maxima**

| *Cluster no. (voxel no.)*  *Region* | *Side* | *MNI*  *X* | *Y* | *Z* | *t-statistic* |
| --- | --- | --- | --- | --- | --- |
| 1 (36432)  Superior longitudinal fascicle  Inferior fronto-occipital fascicle | R  R  R  R  R  R  R | 42  49  43  47  53  42  29 | -3  -10  0  -3  -6  5  18 | 25  25  23  23  20  19  3 | 3.47  3.09  3.26  3.41  3.12  3.11  3.09 |
